# Meta-analysis of GWAS on both Chinese and European populations identifies *GPR173* as a novel X chromosome susceptibility gene for SLE

**DOI:** 10.1186/s13075-018-1590-3

**Published:** 2018-05-03

**Authors:** Huoru Zhang, Yan Zhang, Yong-Fei Wang, David Morris, Nattiya Hirankarn, Yujun Sheng, Jiangshan Shen, Hai-Feng Pan, Jing Yang, Sen Yang, Yong Cui, Dong-Qing Ye, Timothy J. Vyse, Xuejun Zhang, Yu Lung Lau, Wanling Yang

**Affiliations:** 1 Department of Paediatrics and Adolescent Medicine, Queen Mary Hospital, Li Ka Shing Faculty of Medicine, The University of Hong Kong, 21 Sassoon Road, Sandy Bay, Hong Kong; 2 Department of Pediatric Surgery, Guangzhou Institute of Pediatrics, Guangzhou Women and Children’s Medical Center, Guangzhou Medical University, Guangdong, China; 3 Division of Genetics and Molecular Medicine, King’s College London, London, SE1 9RT UK; 4 Lupus Research Unit, Department of Microbiology, Faculty of Medicine, Chulalongkorn University, Bangkok, Thailand; 5 Key Laboratory of Dermatology, Ministry of Education, Anhui Medical University, Hefei, Anhui China; 6 Department of Epidemiology and Biostatistics, School of Public Health, Anhui Medical University, Hefei, Anhui China; 7 Department of Dermatology, China-Japan Friendship Hospital, Beijing, China; 8 Centre for Genomic Sciences, Li Ka Shing Faculty of Medicine, The University of Hong Kong, Sandy Bay, Hong Kong

**Keywords:** Systemic lupus erythematosus, X chromosome, Association, Genetics, Single-nucleotide polymorphisms

## Abstract

**Background:**

Systemic lupus erythematous (SLE) is a complex autoimmune disease with female predominance, particularly affecting those of childbearing age. We performed analysis of three genome-wide genotyping datasets of populations of both Chinese and European origin.

**Methods:**

This study involved 5695 cases and 10,357 controls in the discovery stage. The lead signal on chromosome X was followed by replication in three additional Asian cohorts, with 2300 cases and 4244 controls in total. Conditional analysis of the known associated loci on chromosome X was also performed to further explore independent signals.

**Results:**

Single-nucleotide polymorphism rs13440883 in *GPR173* was found to be significantly associated with SLE (*P*_meta_ = 7.53 × 10^− 9^, OR_meta_= 1.16), whereas conditional analysis provided evidence of a potential independent signal in the *L1CAM-IRAK1-MECP2* region in Asian populations (rs5987175 [*LCA10*]).

**Conclusions:**

We identified a novel SLE susceptibility locus on the X chromosome. This finding emphasizes the importance of the X chromosome in disease pathogenesis and highlights the role of sex chromosomes in the female bias of SLE.

**Electronic supplementary material:**

The online version of this article (10.1186/s13075-018-1590-3) contains supplementary material, which is available to authorized users.

## Background

Systemic lupus erythematosus (SLE) is a systemic autoimmune disorder that leads to autoantibody production and multiorgan damage. Both genetic and environmental components are known to contribute to the disease. SLE demonstrates a dramatic sex bias and predominantly affects women of childbearing age, with an approximately 9:1 female-to-male ratio.

Sex hormones probably play a vital role in the female predominance of the disease. In addition, premenstrual and postmenopausal women show a higher prevalence of SLE than men of an identical age group [1, 2], suggesting mechanisms for the disease other than hormone modulation. Interestingly, an association between SLE and Klinefelter’s syndrome has been reported, and 47,XXY male subjects showed a risk of developing SLE comparable to that of 46,XX females [3], suggesting that the extra copy of the X chromosome in females may be crucial in SLE pathogenesis.

Because the extra copy of chromosome X in females is normally inactivated, the mechanism through which it affects SLE prevalence remains unclear. In female mammals, one of the two copies of chromosome X is silenced by X chromosome inactivation [4]. However, about 23% of the genes on the inactivated X chromosome escape inactivation [5]. Presumably, the risk alleles escaping inactivation would have a higher dose in females than in males. In addition, the skewed X chromosome inactivation (loss of mosaicism) occurs when inactivation of one X chromosome is favored over the other. Through this mechanism, the risk allele may be expressed in more than half of the female cells, contributing to a higher dose in females. It is known that X chromosome genes are silenced through methylation [6]. DNA methylation of CD4^+^ T cells was found to be defective in patients with SLE, and several SLE-associated X chromosomal genes and microRNAs were found to be overexpressed in T cells of female patients with SLE [7]. These observations suggest that the abnormal demethylation may have led to higher expression of the SLE-associated genes and microRNAs, especially in female patients with SLE.

Several susceptibility loci on chromosome X have been reported to be associated with SLE. A single-nucleotide polymorphism (SNP) on the 3′ untranslated region of *TLR7* was found to be associated with SLE in Chinese and Japanese populations, with a higher genetic effect size in males than in females [8]. We previously performed a meta-analysis of genome-wide association study (GWAS) data derived from two Chinese cohorts, including 1659 cases and 3398 controls, and identified another novel variant (rs7062536) located in *PRPS2* as being associated with SLE in Asians [9]. More recently, a study in European populations identified a prominent signal in *CXorf21* [10] on chromosome X as being associated with SLE, and a study on multiple Chinese cohorts identified *LINC01420* [11] as being associated with the disease.

The *L1CAM- IRAK1-MECP2* region, which is a highly complex region spanning ten genes (*L1CAM*, *LCA10*, *AVPR2*, *ARHGAP4*, *NAA10*, *RENBP*, *HCFC1*, *TMEM187*, *IRAK1*, and *MECP2*) has consistently been identified as being associated with SLE susceptibility in different studies [9–12]. Fine mapping by Kaufman et al. identified rs1059702 as the casual variant in the *IRAK1-MECP2* region, whereas the neighboring *L1CAM-NAA10-TMEM187* region showed distinct signals in multiple populations [12]. In our previous Asian study, we also identified two signals in the *L1CAM-TMEM187* region (rs2071128 on *NAA10* and rs17422 on *TMEM187*) as being independently associated with SLE [9]. However, the independent signals across this big region in different populations are not fully understood.

In this study, making use of GWAS data derived from both Chinese and European populations, we performed a cross-ethnicity meta-analysis and followed up on the best novel signal on the X chromosome with analysis in additional independent cohorts. We identified rs13440883 in *GPR173* as a novel X-linked locus associated with SLE. Taking advantage of the increased sample size, we also conducted conditional analysis on the known X-linked SLE loci and identified a potential independent signal in the *L1CAM-IRAK1-MECP2* region. These findings improve the understanding of the role of the X chromosome in this prototypical autoimmune disease that predominantly affects women.

## Methods

### GWAS cohorts

The discovery panel in the present study includes two cohorts with Chinese origin and one cohort with European origin (Table 1). All cases used in this study fulfilled the revised criteria of American College of Rheumatology for SLE. Informed consent was given by all individuals involved. All studies were approved by the corresponding institutional review boards.

**Table 1:** Sample information for discovery panel and replication panel

Sample	Discovery panel			Replication panel		
	HK_GWAS	AH_GWAS	UK_GWAS	HK_REP	TH_REP	AH_REP
	Female	Female	Female	Female	Female	Female
Cases	562	984	3671	738	433	1025
Controls	1274	532	4174	952	699	946
	Male	Male	Male	Male	Male	Male
Cases	50	63	365	NA	27	77
Controls	919	673	2785	NA	266	1381

The discovery panel includes data from three genome-wide association studies (GWAS): Hong Kong (HK_GWAS), Anhui Province (AH_GWAS), and the United Kingdom (UK_GWAS). The replication panel consists of three cohorts: Hong Kong (HK_REP), Thailand (TH_REP), and Anhui Province (AH_REP). In all six datasets, females comprised around 90% of the patients, which is consistent with the female predominance for systemic lupus erythematosus

### Genome-wide genotyping and quality control

The genome-wide genotyping data from Hong Kong [13] and Anhui Province, China [14], were generated using the Human610-Quad BeadChip array (620,901 markers; Illumina, San Diego, CA, USA), whereas the UK GWAS [10] was conducted using the HumanOmni1-Quad BeadChip array (1,140,419 markers; Illumina) and the HumanOmni2.5 BeadChip array (2,443,179 markers; Illumina). Quality control on the X chromosome was conducted in all datasets according to the following procedure. SNPs with a genotyping call rate < 90%, a minor allele frequency < 1%, or violating Hardy-Weinberg equilibrium (*P* < 1 × 10^− 4^ in female controls) were discarded. Individuals with ambiguous gender or with SNP calling rate < 90% were also excluded.

### Imputation

On the basis of genotyping data, we imputed the X chromosome SNPs for all three datasets. First, SHAPEIT [15] was used to prephase each of the datasets. Subsequently, in order to obtain genotypes of additional SNPs, imputation on X chromosome SNPs was performed using IMPUTE v2.3.2 [16] on the three studies separately, using samples from the 1000 Genomes Project (phase 3, released in October 2014, build 37) as the reference. Both of the programs have a specialized algorithm to deal with X chromosome data. In all the studies, SNPs with an imputation score < 0.9 were removed from further analysis.

### Association analysis

The X chromosomal SNPs passing quality control were analyzed for association using SNPTEST [16], fitting a logistic regression model in males and females separately. The association for European data, which comprise samples derived from different cohorts [10], was adjusted using four principal components. The two Asian cohorts were adjusted by the top two principal components. Meta-analysis was conducted using METAL [17], which employs a method based on inverse variance and weights the effect size estimates of each SNP by its SE. For each SNP, Cochran’s *Q* statistic and the *I*^2^ index were used to test for any evidence of genetic heterogeneity between the Chinese and European data.

### Replication in three additional Asian cohorts

After meta-analysis, the top novel SNP with prominent association signal was selected for replication in three different cohorts, including 738 cases and 952 controls from Hong Kong (HK replication panel [HK_REP]), 460 cases and 965 controls from Thailand (Thailand replication panel [TH_REP]), and 1102 cases and 2327 controls from Anhui (Anhui replication panel [AH_REP]) (Table 1). The replication was performed using a TaqMan SNP genotyping assay (Thermo Fisher Scientific, Waltham, MA, USA). The missing genotype rate was less than 10% and was similar between cases and controls.

### Detecting independent signals in known loci on the X chromosome

All of the known SLE risk loci in the X chromosome identified in previous association studies, as well as the SNPs in the surrounding regions (± 200 kb), were closely examined. Pairwise linkage disequilibrium (LD) among the SNPs was calculated to detect any potential independent signals. SNPs with association *P*_meta_ < 1 × 10^− 3^ and low LD with the reported SNP (*r*^2^ < 0.5) were selected for further examination, and the SNPs with a conditional *P* value less than 0.01 were considered as potential independent signals.

Because the association signals in the *L1CAM- MECP2* region were much stronger than the others, the analysis was different for this region. Eighty-eight SNPs with genome-wide significance (*P* < 5 × 10^− 8^) were selected, instead of using the *P* < 1 × 10^− 3^ threshold used in the other regions. An LD block was plotted, and the top SNPs in each block were selected for a conditional analysis to test for independent associations. Only the SNPs with a conditional *P* value less than 0.01 were considered as potentially independent.

### Average genetic risk score

In order to measure the disease risk for SLE for each individual and compare it between the sexes and populations, we performed a modified calculation of genetic risk scores described by Hughes et al*.* [18]. Based on the 63 SLE autosomal susceptibility SNPs characterized by Morris et al*.* [19], a genetic risk score was calculated using the effect size of the risk alleles and the number of copies carried by each individual. Specifically, as shown in eq. (1), the number of risk alleles for SNP *i* (*n*_i_) is multiplied by the natural logarithm of the corresponding OR, which is then summed and divided by *k* (total number of risk SNPs available for this individual) to obtain the average genetic risk score (aGRS):

1 $$ average\ gentic\ risk\ score=\frac{\ {\sum}_{i=1}^k\ln \left({OR}_i\right){n}_i}{k} $$

A total of 1659 Asian SLE samples (Hong Kong and Anhui combined) and a total of 4036 European SLE samples were included to calculate the aGRS, and the differences between male and female cases were analyzed using Student’s *t* test.

### Functional annotation of the susceptibility variants

We used the intragenomic replicates (IGR) method described by Cowper-Sal lari et al*.* [20] to predict the functional impact of a single-nucleotide variant (SNV) on transcription factor (TF) binding. This method takes a 7-bp short DNA sequence (7-mer) containing the target SNV (the 7-mer containing the reference allele is then referred to as “reference 7-mer,” and the 7-mer containing the alternative allele is then referred to as “alternative 7-mer”) and does genome-wide searches for the reference 7-mers and the alternative 7-mers. Then it compares the average chromatin immunoprecipitation sequencing (ChIP-seq) signal intensity of all reference 7-mer matches and all alternative 7-mer matches. A sliding window was used to find all 7-bp short DNA sequences containing the SNV. The reference 7-mer with the highest average intensity and the alternative 7-mer with the highest average intensity would be used for final comparison. All of the matches genome-wide would be filtered to exclude sites outside open chromatin (marked by DNase I hypersensitivity site [DHS]). Two cell lines—GM12878 (lymphoblastoid cell line) and K562 (human immortalized myelogenous leukemia cells)—were used in this IGR analysis. For the TF data in the GM12878 cell line, the corresponding GM12878 DHS data were used as the filter, and for the TF data in K562 cell line, the K562 DHS data were used as the filter. ChIP-seq files used in the analysis were downloaded from the ENCODE [21] website, and a complete list of files is provided in Additional file 1: Table S1.

## Results

### X chromosome meta-analysis

**Fig. 1:**
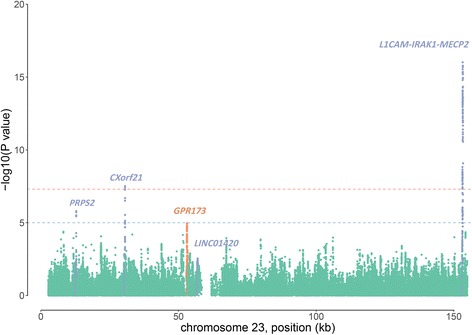
Manhattan plot of the X chromosome meta-analysis results of three genome-wide association studies. The *x*-axis is position (kb) on the X chromosome, and the *y*-axis is the *P* value (− log_10_ *P*) for cross-population X chromosome meta-analysis. Known systemic lupus erythematosus susceptibility genes are labeled in *blue*, and the gene including the novel loci (rs13440883) is labeled in *red*. The *dashed lines* indicate the suggestive *P* value (1 × 10^− 5^, *blue*) and the genome-wide significant *P* value (5 × 10^− 8^, *red*)

Our study consisted of three sets of GWAS data, including two GWASs from China [13, 14] and one from the United Kingdom [10] (Table 1). Considering the sex difference in X chromosome dosage, we analyzed the female and male samples separately. After imputation by IMPUTE2 [16] and association analysis by SNPTEST [15], meta-analysis of the six datasets (female and male being analyzed separately) was conducted by METAL [17] using an inverse-variance-based method. A quantile-quantile plot (Additional file 1: Figure S1) was generated to evaluate association signals on the X chromosome. After removing all SNPs within the known X-linked SLE susceptibility loci and those within the range of ± 200 kb, the data (Additional file 1: Figure S1b) still deviated from the null expectation, which suggests that there are more novel X-linked SLE susceptibility loci to be discovered. A Manhattan plot (Fig. 1) was also generated to gain a systematic view of the association signals. Apart from the reported X-linked SLE susceptibility loci and the genes to which they are mapped, there seemed to be more novel signals to be further investigated.

### Replication and identification of novel SLE susceptibility loci

On the basis of our meta-analysis results, after removing known associated regions as mentioned above, 48 SNPs with *P*_meta_ < 1 × 10^− 4^ level of significance were further analyzed (Additional file 1: Table S2). Pruning based on LD (*r*^2^ < 0.3) excluded 39 SNPs from the list, leaving 9 SNPs with potential independent signals (Additional file 1: Table S3). Among them, SNP rs13440883 showed the most prominent signal. LocusZoom [22] was used to plot the regional (± 200 kb) association signal (Additional file 1: Figure S2). The association of rs13440883 was detected in both Asian and European data, with a slightly higher effect size in Asians (OR_HK_ = 1.18; OR_AH_ = 1.18; OR_EUR_ = 1.13).

**Fig. 2:**
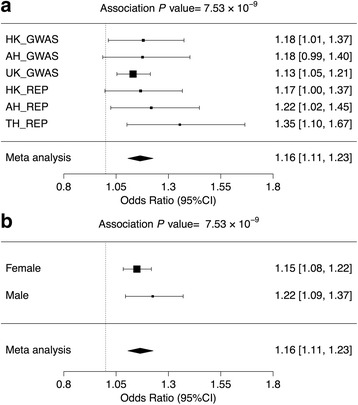
Forest plot showing ORs for rs13440883 in *GPR173*. **a** ORs of different cohorts. **b** ORs of the two sexes. *HK_GWAS* Hong Kong genome-wide association studies, *AH_GWAS* Anhui Province genome-wide association studies, *UK_GWAS* United Kingdom genome-wide association studies, *HK_REP* Hong Kong replication panel, *TH_REP* Thailand replication panel, *AH_REP* Anhui Province replication panel

SNP rs13440883, as the most prominent signal, was selected for further replication. Three independent cohorts were used in the replication, with a total of 2300 cases and 4244 controls. After analysis of the replication results together with the GWAS data, the selected SNP (rs13440883) showed a final *P*_meta_ value of 7.53 × 10^− 9^, reaching genome-wide significance (Fig. 2).

Conditional analyses were performed on Asian and European GWAS data to test for potential independent signals in this locus. Within the ± 200-kb window, there were 60 SNPs with association signals (*P*_meta_ < 1 × 10^− 3^). However, after conditional analysis, none of them remained significant. Thus, based on the samples available, rs13440883 is the only independent association signal confirmed in this region.

### Investigation of underlying mechanism for the novel replicated locus

Data on histone modification, DNase hypersensitivity, and TF binding, accessible using ENCODE [21], were used to identify functionally important SNVs, and rs13440883 was found to be within a region marked by H3K27ac and H3K4me1 in CD19 primary cells. The SNPs in high LD (*r*^2^ > 0.8) with rs13440883 in both European and Asian populations and different TFs with binding peaks overlapping the corresponding SNPs are highlighted in Additional file 1: Figure S3a. Among them, SNP rs11091720, which showed high LD with rs13440883 (*r*^2^_EUR_ = 0.9384; *r*^2^_Asian_ = 0.9915), was a TF binding hot spot in the coding region of *GPR173*.

The IntraGenomic Replicates tool [20] was used to predict potential TF binding affinity differences that may lead to differential gene expression for both rs13440883 and rs11091720 (Additional file 1: Figure S3b and c). For rs13440883, the prediction showed a significant increase (*P* = 3.54 × 10^− 5^) in the chromatin-binding intensity of SMARCA4 for the alternative allele. For rs11091720, the prediction showed a significant decrease in the intensity of L3MBTL2 (*P* = 2.31 × 10^− 13^) and CTCFL (*P* = 3.67 × 10^− 10^), as well as an increase in the intensity of CTCF (*P* = 6.22 × 10^− 11^) and JUN (*P* = 0.0064) for the alternative allele. SMARCA4 is a component of a large ATP-dependent chromatin-remodeling complex (SNF/SWI) that is required for transcriptional activation of genes normally repressed by chromatin. CTCF forms methylation-sensitive insulators that may regulate X chromosome inactivation. The function of L3MBTL2, CTCFL, and JUN was not previously known. However, before experimental validation, we had to treat the results of this in silico analysis carefully, which is discussed later.

### Identification of independent signals in the known loci on the X chromosome

We further examined the associations of the reported susceptibility loci in previous studies, including *PRPS2*, *LINCO1420*, *CXorf21*, and *IRAK1*. The susceptibility gene *TLR7* is not examined in this analysis due to poor coverage of this region. All the SNPs within a ± 200-kb window centered on the reported SNPs were filtered by association *P* values and pairwise LD (*r*^2^) with each other (*see* detailed criteria in the Methods section above) before analysis of independence. For the first three loci, we found no SNP passing the filtering criteria.

Meta-analysis of the GWAS datasets showed 88 SNPs in the *L1CAM-MECP2* region (153,284,192 ± 200 kb, hg19) attained genome-wide significance (*P* < 5 × 10^− 8^) (Additional file 1: Table S4), including the previously reported SNPs [9]. Independent contributions of each risk-associated SNP in this region was further examined. The LD pattern of the 88 SNPs was plotted separately for Anhui, Hong Kong, and U.K. data (Additional file 1: Figure S4). In the two Asian datasets, similar patterns were found, and four LD blocks were observed. The top SNPs from each block were then selected for a conditional test (Additional file 1: Table S5). We noted that rs5987175 (*P*_meta_ = 1.50 × 10^− 9^) in *LCA10* exhibited an independent contribution toward SLE susceptibility. After adjusting for the effect of the known independent SNPs (rs1059702, rs17422, rs2071128) reported in our previous study [9], rs5987175 was still significant in Asians (Table 2). However, the independence could not be replicated in the U.K. cohort, probably owing to higher LD between the blocks in Europeans, which is discussed later.

**Table 2:** Association *P* values for rs5987175 after adjusting for effect of known independent single-nucleotide polymorphisms in Asian populations

SNPs added as covariates	*P*_Asian_	OR_Asian_ with CI
None	1.66 × 10^− 5^	1.28 (1.14, 1.43)
rs2071128	7.32 × 10^− 3^	1.19 (1.05, 1.35)
rs17422	2.83 × 10^−3^	1.19 (1.06, 1.35)
rs1059702	6.53 × 10^−3^	1.18 (1.05, 1.33)
rs2071128, rs17422	6.98 × 10^−3^	1.19 (1.05, 1.36)
rs2071128, rs1059702	6.65 × 10^−3^	1.20 (1.05, 1.36)
rs17422, rs1059702	8.79 × 10^−3^	1.17 (1.04, 1.33)
rs17422, rs2071128, rs1059702	6.55 × 10^−3^	1.20 (1.05, 1.36)

*SNP* Single-nucleotide polymorphism

## Discussion

In this cross-population meta-analysis of three GWAS datasets and further replication in three additional cohorts, with a total of 7995 cases and 14,601 healthy controls, we successfully identified a novel variant (rs13440883, *P*_*meta*_ = 7.53 × 10^− 9^, OR_meta_ = 1.16) within *GPR173*, as well as a potential independent signal (rs5987175, *P* = 6.55 × 10^− 3^, upon adjusting for the effect of rs17422, rs2071128, and rs1059702 together in Asians) within *LCA10* of the *L1CAM-MECP2* region on chromosome X as being associated with SLE.

The novel risk-associated variant, rs13440883, is located within the intron between the second and third exons of *GPR173*. According to the regulatory annotation data provided by the ENCODE [21] project (Additional file 1: Figure S3a), this SNP lies within a DNase I-hypersensitive site detected in CD19 primary cells, CD4^+^ naive Wb78495824, and mobilized CD56 primary cells. It is also within the binding site of SMARCA4 detected in a K562 cell line. Analysis using the IntraGenomic Replicates tool [20] predicted that the alternative allele would have a 1.49-fold higher binding intensity for this TF, which is a significant increase (*P* = 3.54 × 10^− 5^ by Student’s *t* test). SMARCA4 is involved in the glucocorticoid receptor regulatory network, which was reported to affect sex differences in the prevalence of inflammatory disease [23]. However, although the IntraGenomic Replicates tool implemented multiple methods to avoid false-positive results, it must be borne in mind that this tool gives a prediction based only on in silico experiments, and the TF binding alteration still needs to be confirmed by further experiments.

Among all the disease- or trait-associated variants detected by GWAS, a majority are located in noncoding regions and enriched in regulatory DNA sequences marked by DNase I-hypersensitive sites [24]. For most associations, the SNP most strongly supported by functional annotation is often the one in high LD with the reported SNP [25]. In the present study, although the RegulomeDB [26] score for rs13440883 is only 3a (less likely to affect binding), the score for rs11091720, which is in perfect LD with rs13440883, is 2c (likely to affect binding), which suggests that this SNP might be more important in terms of function. Among all the SNPs in high LD (*r*^2^ > 0.8) with rs13440883 detected in the present study, rs11091720 is the only one located in the coding region of *GPR173* (synonymous). It is also the only one with multiple TF binding data (Additional file 1: Figure S3a). CTCF, which is a well-known TF binding insulator, was found to bind to the region containing rs11091720 in multiple cell lines, including GM12878, other lymphoblastoid cell lines, K562, HeLa S3, and many others. IGR [20] predicted an increased CTCF binding intensity for the alternative allele, which may perturb the effect of the insulator and could potentially be another functional explanation. Again, the IGR prediction still needs to be validated, and further studies are still needed to clarify whether this alteration would lead to disease susceptibility.

Although there is as yet no existing evidence showing their influence on the gene’s expression, both rs13440883 and rs11091720 are located within the *GPR173* gene, which encodes G protein-coupled receptor (GPCR) 173, a member of the G protein-coupled receptor 1 family. In our previous study, an SNP in the upstream of *GPR19* was identified as an SLE susceptibility [13] variant. There are also some other GPCR-encoding genes reported to be associated with autoimmune diseases [27–32].

Consistent with previous studies on the X chromosome, the strongest association signal in our meta-analysis was from the *L1CAM-MECP2* region, with 88 SNPs reaching genome-wide significance in the discovery panel alone. A conditional test using logistic regression was performed to test for independent signals, and rs5987175 in *LCA10* was found to remain significant in Asians, after adjusting for the effect of three known independent SNPs (rs1059702, rs17422, and rs2071128) (Table 2). However, in the European dataset, the LD between rs5987175 and other SNPs (rs17422, *r*^2^ = 0.41; rs1059702, *r*^2^ = 0.33) was higher than in Asians (rs17422, *r*^2^ = 0.12; rs1059702, *r*^2^ = 0.09). Thus, a larger sample size would be needed to acquire adequate power to assess independent signals in this region in Europeans.

**Fig. 3:**
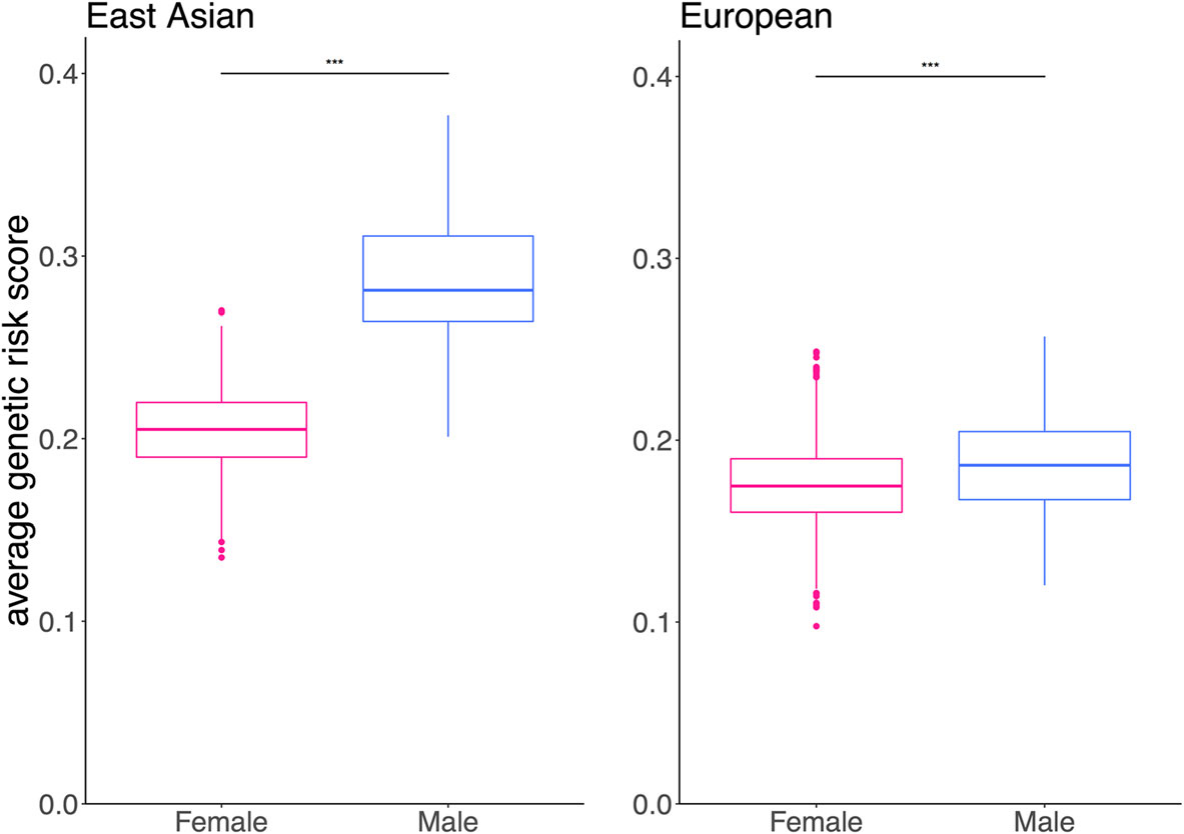
Average genetic risk score. The average genetic risk score (GRS) was calculated separately for Asian females, Asian males, European females, and European males. Evidence of differences in GRSs was detected between different sexes in both Asian and European populations (Asian females vs males, *P* = 2.2 × 10^− 16^; European females vs males, *P* = 8.295 × 10^− 7^). The finding that male patients showed a higher disease burden than female patients is consistent with previous results, suggesting that in different populations, the genetic effect size required in male patients is higher than in female patients (***P < 0.001)

The novel association signal detected in the present study has a larger effect size in males than in females (Fig. 2b), which is consistent with previous X-linked studies [8, 9], and again suggests that these X-linked variants are not involved in escaping X inactivation and do not contribute to the female bias of SLE. The aGRS for SLE was also calculated, with 63 SNPs identified in autosomes. Not only do Asians have a higher GRS than Europeans, but males also have a higher GRS than females in the same population (statistically significant in both Asian and European population) (Fig. 3), which is consistent with the previous Asian-only study [9] that included 32 SNPs in the calculation. This result might indicate that a relatively higher genetic predisposition is required for males to develop SLE.

## Conclusions

In our present cross-population meta-analysis following replication, we identified a novel SLE-associated variant (rs13440883) in *GPR173* and a potential independent signal (rs5987175) in *LCA10.* Further functional annotation and in silico analysis provided plausible candidates for functional causal variants, although experiments are still needed to validate the prediction. A larger sample size is needed to further confirm the independence of rs5987175 in Europeans. The fact that males have a higher effect size than females indicates that the currently identified X-linked SNP is probably not contributing to the female prevalence of the disease. Further investigations are still needed to elucidate the mechanism of the sex bias of SLE.

## Additional file

Additional file 1: **Table S1.** Complete list of ChIP-seq files used in Intragenomic Replicates (IGR) analysis. **Table S2.** List of the 48 SNPs with association P value smaller than 1 × 10^-4^. **Table S3.** List of the candidate X-linked SLE susceptibility genes. **Table S4.** List of the 88 SNPs surpassing genome wide significance in L1CAM-MECP2 region. **Table S5.** Conditional logistic regression results in both Asian GWAS and European GWAS. **Table S6.** The list of SNVs used in Figure S3A. **Figure S1.** QQ plot for the cross-population X chromosome meta-analysis data. **Figure S2**. The LocusZoom Plot showing association significance and local LD for the region around rs13440883 (±200kb). **Figure S3.** Identification of functional risk-associated SNV shared between Europeans and Asians. **Figure S4.** LD patterns of the risk-associated SNPs in L1CAM-MECP2 region. (PDF 2421 kb)

## Abbreviations

aGRS: Average genetic risk score
ChIP-seq: Chromatin immunoprecipitation sequencing
DHS: DNase I hypersensitivity site
GPCR: G protein-coupled receptor
GWAS: Genome-wide association study
IGR: Intragenomic replicates
LD: Linkage disequilibrium
SLE: Systemic lupus erythematosus
SNP: Single-nucleotide polymorphism
SNV: Single-nucleotide variant
TF: Transcription factor
