# Supplementary material for: Meta-analysis of GWAS on both Chinese and European populations identifies GPR173 as a novel X chromosome susceptibility gene for SLE
Source: Arthritis Res Ther. 2018 May 3;20:92. doi: 10.1186/s13075-018-1590-3 (PMC5934841; doi:10.1186/s13075-018-1590-3)
Supplement: Supplementary file 1 — Table S1. Complete list of ChIP-seq files used in Intragenomic Replicates (IGR) analysis. Table S2. List of the 48 SNPs with association P value smaller than 1 × 10^-4^. Table S3. List of the candidate X-linked SLE susceptibility genes. Table S4. List of the 88 SNPs surpassing genome wide significance in L1CAM-MECP2 region. Table S5. Conditional logistic regression results in both Asian GWAS and European GWAS. Table S6. The list of SNVs used in Figure S3A. Figure S1. QQ plot for the cross-population X chromosome meta-analysis data. Figure S2. The LocusZoom Plot showing association significance and local LD for the region around rs13440883 (±200kb). Figure S3. Identification of functional risk-associated SNV shared between Europeans and Asians. Figure S4. LD patterns of the risk-associated SNPs in L1CAM-MECP2 region. (PDF 2421 kb) [file 13075_2018_1590_MOESM1_ESM.pdf]

Supplementary Tables

Supplementary Table 1. Complete list of ChIP-seq files used in Intragenomic Replicates (IGR) analysis.

ChIP-seq files for transcription factor binding	Transcription factor	Cell line
ENCFF225MPC.bed	ARID1B	K562
ENCFF002DDJ.bed	CTCF	K562
ENCFF002CLT.bed	CTCFL	K562
ENCFF002CMA.bed	GATA2	K562
ENCFF185QPE.bed	HDAC1	K562
ENCFF002CWC.bed	JUN	K562
ENCFF738WCE.bed	KDM1A	K562
ENCFF468PSZ.bed	L3MBTL2	K562
ENCFF986MHU.bed	NFXL1	K562
ENCFF657YIC.bed	NRF1	K562
ENCFF002CWK.bed	RCOR1	K562
ENCFF197YHU.bed	SMARCA4	K562
ENCFF002CYH.bed	TAL1	K562
ENCFF447QUG.bed	ZNF512	K562
ENCFF002COK.bed	BHLHE40	GM12878
ENCFF002COQ.bed	CTCF	GM12878
ENCFF002COR.bed	E2F4	GM12878

ENCFF002CGW.bed	EGR1	GM12878
ENCFF002CGX.bed	ELF1	GM12878
ENCFF002COT.bed	ELK1	GM12878
ENCFF002COW.bed	MAX	GM12878
ENCFF002COX.bed	MAZ	GM12878
ENCFF002CPA.bed	RELA	GM12878
ENCFF002CPD.bed	NRF1	GM12878
ENCFF002CPH.bed	POLR2A	GM12878
ENCFF002CHP.bed	POU2F2	GM12878
ENCFF002CPK.bed	RAD21	GM12878
ENCFF002CHS.bed	RUNX3	GM12878
ENCFF002CPN.bed	SMC3	GM12878
ENCFF002CHV.bed	SP1	GM12878
ENCFF002CHW.bed	SRF	GM12878
ENCFF002CHY.bed	TAF1	GM12878
ENCFF812DEY.bed	TCF7L2	K562
ENCFF002CIC.bed	YY1	GM12878
ENCFF002CPW.bed	ZNF143	GM12878
ChIP-seq files for DHS		
wgEncodeUwDnaseK562PkRep1.narrowPeak	Not applicable	K562
wgEncodeUwDnaseGm12878PkRep1.narrowPeak	Not applicable	GM12878

Supplementary Table 2. List of the 48 SNPs with association P_{gwas} value smaller than 1E-04, after removing the previously reported SNPs and the SNPs surrounding them (±200 kb). The novel signal (rs13440883) reported in the current study is labeled in bold.

Gene	Function	SNP	Position	Direction	P_{gwas}	OR_{gwas}	P_{adj}	OR_{adj}	I^2	Het*P*Val
VCX2, VCX3B	intergenic	rs111682547	8183477	---+??	3.14E-05	0.59	4.83E-05	0.60	0	0.50
VCX2, VCX3B	intergenic	rs145425115	8211854	+++-??	3.16E-05	1.69	4.20E-05	1.68	0	0.45
MSL3, FRMPD4	intergenic	rs6640789	11857936	----??	4.68E-05	0.81	1.16E-04	0.81	0	0.83
YY2	UTR5	rs2382759	21874387	---+--	6.67E-05	0.90	8.44E-05	0.90	29.3	0.22
MBTPS2	UTR3	rs5951476	21901903	++--++	6.34E-05	1.11	8.75E-05	1.11	40.4	0.14
DMD	intronic	rs5928194	33207009	??++??	5.55E-05	1.34	6.45E-05	1.34	0	0.89
MAGED1	intronic	rs12863962	51581783	????--	6.93E-05	0.83	6.93E-05	0.83	0	0.50
FAM156A, GPR173	intergenic	rs12011862	53059319	++++++	8.15E-05	1.12	8.11E-05	1.12	0	0.72
FAM156A, GPR173	intergenic	rs68049065	53060045	------	9.56E-05	0.89	1.03E-04	0.89	0	0.57
FAM156A, GPR173	intergenic	rs12014182	53065809	---+--	6.61E-05	0.89	7.21E-05	0.89	0	0.56
FAM156A, GPR173	intergenic	rs12400146	53071436	+++-++	5.64E-05	1.13	5.97E-05	1.13	0	0.61
GPR173	intronic	rs55850908	53081414	------	1.10E-05	0.88	1.70E-05	0.88	0	0.69
GPR173	intronic	rs59180338	53081558	------	1.08E-05	0.87	1.66E-05	0.88	0	0.69
GPR173	intronic	rs67791620	53081814	------	7.13E-05	0.88	9.17E-05	0.88	0	0.69
GPR173	intronic	rs67450314	53081970	------	1.45E-05	0.88	2.13E-05	0.88	0	0.69
GPR173	intronic	rs12392162	53082363	------	1.70E-05	0.88	2.50E-05	0.88	0	0.66
GPR173	intronic	rs12007081	53084492	------	2.07E-05	0.88	3.09E-05	0.88	0	0.67
GPR173	intronic	rs12013318	53084568	++++++	2.42E-05	1.14	3.60E-05	1.13	0	0.68
GPR173	intronic	rs7473078	53084799	------	2.07E-05	0.88	3.09E-05	0.88	0	0.67
GPR173	intronic	rs67457527	53085169	++++++	1.72E-05	1.14	2.58E-05	1.14	0	0.69
GPR173	intronic	rs12389147	53086749	++++++	1.70E-05	1.14	2.42E-05	1.14	0	0.86

GPR173	intronic	rs12390489	53087476	++++++	2.46E-05	1.14	3.80E-05	1.13	0	0.76
GPR173	intronic	rs67171574	53087835	------	1.25E-05	0.88	1.97E-05	0.88	0	0.79
GPR173	intronic	rs189101417	53089213	++++++	4.56E-05	1.13	7.33E-05	1.13	0	0.65
GPR173	intronic	rs10855213	53090536	++++++	1.59E-05	1.14	2.59E-05	1.13	0	0.71
GPR173	intronic	rs73634281	53095639	------	1.12E-05	0.88	1.86E-05	0.88	0	0.73
GPR173	intronic	rs67733752	53096176	------	1.07E-05	0.88	1.78E-05	0.88	0	0.72
GPR173	intronic	rs28585589	53098061	---+--	8.17E-05	0.88	1.43E-04	0.88	0	0.84
GPR173	intronic	rs111655908	53098327	+++-++	8.52E-05	1.14	1.50E-04	1.13	0	0.84
GPR173	intronic	rs112167847	53100196	---+--	4.60E-05	0.88	8.17E-05	0.88	0	0.84
GPR173	intronic	rs12389413	53100590	------	1.06E-05	0.88	1.79E-05	0.88	0	0.74
GPR173	intronic	rs73634282	53101228	++++++	1.04E-05	1.14	1.73E-05	1.14	0	0.76
GPR173	**intronic**	**rs13440883**	**53101477**	**++++++**	**7.74E-06**	**1.15**	**1.25E-05**	**1.14**	**0**	**0.83**
GPR173	intronic	rs7881236	53101684	---+--	5.39E-05	0.87	9.21E-05	0.88	0	0.68
GPR173	intronic	rs7883255	53101934	++++++	6.27E-06	1.15	1.10E-05	1.15	0	0.78
GPR173	intronic	rs7473421	53102956	++++++	2.13E-05	1.14	3.72E-05	1.14	0	0.81
GPR173	intronic	rs12388258	53105196	++++++	4.66E-05	1.14	7.14E-05	1.13	0	0.92
GPR173	exonic	rs11091720	53106865	------	6.40E-05	0.88	9.52E-05	0.88	0	0.92
GPR173	UTR3	rs56157110	53107941	------	4.62E-05	0.88	7.15E-05	0.88	0	0.94
RNF128, TBC1D8B	intergenic	rs73247960	106044726	------	9.86E-05	0.90	1.88E-04	0.90	24.5	0.25
TBC1D8B, RIPPLY1	intergenic	rs4409525	106140325	------	5.40E-05	0.90	1.05E-04	0.90	0	0.54
HMGB3, MIR4330	intergenic	rs3866987	150231297	+-++++	7.74E-05	1.11	9.95E-05	1.11	4.8	0.39
F8	intronic	rs6655259	154228944	????--	9.59E-05	0.73	9.59E-05	0.73	0	0.60
F8	intronic	rs113298253	154239725	????--	6.52E-05	0.73	6.52E-05	0.73	0	0.67
FUNDC2	intronic	rs113884477	154276531	????--	4.58E-05	0.72	4.58E-05	0.72	0	0.72
FUNDC2	intronic	rs113240511	154277000	????--	6.41E-05	0.73	6.41E-05	0.73	0	0.65

FUNDC2	intronic	rs111262877	154281330	????--	5.28E-05	0.72	5.28E-05	0.72	0	0.68
FUNDC2	downstream	rs111837982	154285625	????--	7.97E-05	0.73	7.97E-05	0.73	0	0.66

Supplementary Table 3. List of the candidate X-linked SLE susceptibility genes. The SNP (rs13440883) with genome wide significance identified in the current study is labeled in bold.

Gene	Function	SNP	Position	Direction	P_{gwas}	OR_{gwas}	P_{adj}	OR_{adj}	I^2	Het*P*Val
VCX2, VCX3B	intergenic	rs145425115	8211854	+++-??	3.16E-05	1.69	4.20E-05	1.68	0	0.45
MSL3, FRMPD4	intergenic	rs6640789	11857936	----??	4.68E-05	0.81	1.16E-04	0.81	0	0.83
MBTPS2	UTR3	rs5951476	21901903	++--++	6.34E-05	1.11	8.75E-05	1.11	40.4	0.14
DMD	intronic	rs5928194	33207009	??++??	5.55E-05	1.34	6.45E-05	1.34	0	0.89
MAGED1	intronic	rs12863962	51581783	????--	6.93E-05	0.83	6.93E-05	0.83	0	0.50
GPR173	**intronic**	**rs13440883**	**53101477**	**++++++**	**7.74E-06**	**1.15**	**1.25E-05**	**1.14**	**0**	**0.83**
TBC1D8B, RIPPLY1	intergenic	rs4409525	106140325	------	5.40E-05	0.90	1.05E-04	0.90	0	0.54
HMGB3, MIR4330	intergenic	rs3866987	150231297	+-++++	7.74E-05	1.11	9.95E-05	1.11	4.8	0.39
F8	intronic	rs6655259	154228944	????--	9.59E-05	0.73	9.59E-05	0.73	0	0.60

Supplementary Table 4. List of the 88 SNPs surpassing genome wide significance (5E-08) in *L1CAM-MECP2* region. The SNPs are ordered by position and separated by LD block. SNPs used in conditional regression are labeled in bold. Previously identified independent SNPs are labeled in green. And the novel independent SNP rs5987175 is labeled in red.

SNP	Position	Direction	P_{gwas}	OR_{gwas}	P_{adj}	OR_{adj}	I^2	Het*P*Val
rs4646263	153140449	------	7.75E-09	0.85	1.45E-08	0.86	0	0.7315
rs12156710	153150791	------	1.41E-08	0.85	2.23E-08	0.86	22.1	0.2676
rs41311376	153150929	------	6.56E-09	0.85	8.90E-09	0.85	27	0.2318
rs73627240	153151939	------	4.15E-09	0.85	5.44E-09	0.85	0	0.4604
rs73627241	153152126	------	3.95E-09	0.85	5.16E-09	0.85	0	0.4619
rs5945168	153152760	------	5.85E-09	0.85	7.53E-09	0.85	0	0.4942
**rs5987175**	**153153839**	**------**	**1.21E-09**	**0.85**	**1.50E-09**	**0.85**	**16.1**	**0.3099**
rs4898456	153155214	------	2.38E-09	0.85	3.12E-09	0.85	0	0.5429
rs5945365	153156390	++++++	6.24E-09	1.17	8.42E-09	1.17	0	0.6785
rs4898457	153158356	------	2.97E-09	0.85	4.11E-09	0.85	2.8	0.3984
rs5945169	153162037	++++++	7.73E-09	1.17	1.00E-08	1.17	0	0.7636
rs7052686	153162462	------	6.50E-09	0.85	8.59E-09	0.85	0	0.5192
rs7886319	153162986	++++++	1.07E-08	1.17	1.27E-08	1.17	0	0.5866
rs5987179	153163197	++++++	1.80E-08	1.17	2.41E-08	1.16	0	0.4617
rs5945367	153164342	------	1.54E-09	0.85	2.22E-09	0.85	0	0.5416
rs5945368	153165516	------	2.43E-09	0.85	3.64E-09	0.85	0	0.7698
rs5945369	153165804	++++++	2.52E-08	1.17	3.63E-08	1.17	0	0.7147
rs5945370	153165883	------	2.93E-09	0.85	4.39E-09	0.85	0	0.7607
rs2872601	153166009	------	2.53E-09	0.85	3.77E-09	0.85	0	0.7713

rs4898458	153167690	++++++	3.35E-09	1.17	3.66E-09	1.17	0	0.4417
rs3761528	153168449	++++++	3.95E-08	1.16	4.26E-08	1.16	0	0.564
rs2269372	153207545	++++++	7.52E-15	1.27	1.74E-14	1.26	0	0.74
rs2269373	153207925	------	4.20E-15	0.79	1.05E-14	0.79	0	0.76
rs4898465	153208140	------	3.37E-15	0.79	8.68E-15	0.79	0	0.75
rs3027869	153211538	------	2.77E-14	0.79	6.06E-14	0.79	0	0.68
rs762656	153211652	++++++	4.86E-14	1.26	1.08E-13	1.25	0	0.66
rs2071132	153216036	------	3.08E-14	0.8	7.32E-14	0.80	0	0.67
rs3027878	153218365	------	7.13E-16	0.77	1.63E-15	0.77	0	0.49
rs762653	153218920	------	2.29E-16	0.77	4.99E-16	0.77	0	0.58
rs2071133	153219665	------	1.97E-14	0.79	5.42E-14	0.80	0	0.66
rs1051152	153220360	++++++	3.17E-14	1.26	8.20E-14	1.25	0	0.62
rs730106	153221657	++++++	1.78E-14	1.26	4.61E-14	1.25	0	0.58
rs2071134	153222835	------	1.24E-16	0.77	2.88E-16	0.77	0	0.58
**rs17421**	**153225634**	**------**	**3.61E-17**	**0.77**	**9.87E-17**	**0.77**	**0**	**0.74**
rs632	153226471	++++++	2.16E-14	1.26	5.57E-14	1.25	0	0.59
rs17422	153227426	++++++	1.66E-14	1.26	4.42E-14	1.25	0	0.6
rs59607260	153227770	------	1.75E-14	0.79	4.48E-14	0.79	0	0.62
rs2266886	153231352	++++++	1.32E-14	1.26	3.63E-14	1.26	0	0.66
rs6655268	153238288	++++++	8.54E-14	1.26	1.93E-13	1.25	0	0.57
rs2266887	153239587	------	2.64E-14	0.8	6.87E-14	0.80	0	0.61
rs2266888	153239720	------	2.28E-14	0.79	5.84E-14	0.80	0	0.62
rs4898374	153241386	------	1.66E-14	0.79	4.35E-14	0.79	0	0.63
rs5945172	153243315	++++++	8.69E-15	1.26	2.25E-14	1.26	0	0.61

rs12384878	153245128	++++++	5.39E-15	1.27	1.38E-14	1.27	0	0.63
rs113339491	153245217	------	3.58E-15	0.79	9.18E-15	0.79	0	0.64
rs6643653	153246018	++++++	2.07E-14	1.27	5.41E-14	1.26	0	0.61
rs2266890	153247722	------	9.22E-15	0.79	3.66E-14	0.79	0	0.75
rs7350355	153247745	++++++	6.84E-15	1.26	2.80E-14	1.26	0	0.74
rs6571303	153247954	------	7.25E-15	0.79	2.96E-14	0.79	0	0.73
**rs13397**	**153248248**	**------**	**1.34E-16**	**0.77**	**4.75E-16**	**0.77**	**0**	**0.47**
rs5945173	153250172	------	3.08E-14	0.79	1.15E-13	0.80	0	0.73
rs6643808	153252147	++++++	9.60E-15	1.27	4.27E-14	1.26	0	0.66
rs6643809	153252908	++++++	1.95E-14	1.26	8.15E-14	1.26	0	0.66
rs6643656	153254605	++++++	2.29E-13	1.26	6.38E-13	1.26	0	0.69
rs6655269	153256435	------	2.13E-15	0.78	8.22E-15	0.79	0	0.46
rs5986947	153256505	------	1.37E-15	0.78	5.41E-15	0.79	0	0.48
rs5945384	153260414	++++++	8.82E-14	1.26	2.91E-13	1.26	0	0.48
rs11795678	153265728	++++++	2.64E-14	1.27	4.40E-14	1.27	30.3	0.21
rs5945386	153269755	++++++	3.70E-14	1.27	5.86E-14	1.27	35.5	0.17
rs4898375	153273226	------	3.79E-16	0.76	5.26E-16	0.77	22.3	0.27
rs633	153274228	++++++	3.91E-13	1.25	6.72E-13	1.25	40.6	0.13
rs12400188	153275075	++++++	2.80E-13	1.25	4.84E-13	1.25	40.1	0.14
rs3027898	153275890	++++++	8.07E-13	1.24	1.32E-12	1.24	43.5	0.12
rs731642	153277507	------	2.74E-14	0.79	4.38E-14	0.79	39.1	0.14
rs2239673	153277889	++++++	5.65E-13	1.25	9.77E-13	1.25	43.7	0.11
rs763737	153278307	++++++	1.90E-13	1.25	3.32E-13	1.25	40.2	0.14
rs5945174	153279858	++++++	9.25E-14	1.25	1.76E-13	1.25	41.9	0.13

rs7061789	153280475	++++++	8.93E-14	1.25	1.70E-13	1.25	41.6	0.13
**rs1059702**	**153284192**	**------**	**1.12E-16**	**0.76**	**1.84E-16**	**0.76**	**22.8**	**0.26**
rs1059701	153284483	++++++	1.34E-13	1.25	2.57E-13	1.25	43.5	0.12
rs2734647	153292180	------	4.12E-16	0.77	4.40E-16	0.77	5.9	0.38
rs2075596	153297392	------	1.57E-16	0.76	2.04E-16	0.76	3.8	0.39
rs3027933	153298874	++++++	1.16E-16	1.32	1.67E-16	1.32	0	0.5
rs4898467	153299924	++++++	7.80E-12	1.24	1.28E-11	1.23	38	0.15
rs5987194	153301467	------	4.00E-16	0.76	6.50E-16	0.76	0	0.47
rs76613700	153301653	++++++	3.90E-12	1.25	6.23E-12	1.24	27	0.23
rs909131	153308227	++++++	1.11E-11	1.24	1.71E-11	1.24	28.1	0.22
rs17435	153311980	++++++	5.29E-12	1.24	8.41E-12	1.24	23.5	0.26
rs1624766	153317154	++++++	1.25E-12	1.25	2.16E-12	1.25	28	0.22
rs1734787	153325446	++++++	7.97E-16	1.3	1.28E-15	1.30	0	0.43
rs1616369	153326464	------	3.92E-16	0.77	6.30E-16	0.77	3.3	0.4
rs1734791	153330920	++++++	6.98E-16	1.3	1.12E-15	1.30	0	0.44
rs1734789	153331463	++++++	2.21E-12	1.25	4.00E-12	1.24	18.9	0.29
rs1734792	153341060	------	3.53E-15	0.77	4.56E-15	0.77	5.4	0.38
rs2239464	153348431	------	5.34E-13	0.8	9.49E-13	0.80	11.8	0.34
rs5945393	153349428	++++++	6.48E-13	1.25	1.17E-12	1.25	7.5	0.37
rs5986957	153372505	------	1.93E-14	0.78	2.53E-14	0.78	0	0.52
rs2872736	153376436	++++++	1.66E-11	1.24	2.77E-11	1.23	12.7	0.33

Supplementary Table 5. Conditional logistic regression results in both Asian GWAS and European GWAS.

CHR	SNP	BP	conditional on	TEST	Population	OR	*P*
23	rs17421	153225634	rs5987175	ADD	Asian	0.80	1.27E-03
23	rs13397	153248248	rs5987175	ADD	Asian	0.82	7.99E-04
23	rs1059702	153284192	rs5987175	ADD	Asian	0.73	9.21E-06
23	rs5987175	153153839	rs17421	ADD	Asian	0.84	4.55E-03
23	rs5987175	153153839	rs13397	ADD	Asian	0.82	1.18E-03
23	rs5987175	153153839	rs1059702	ADD	Asian	0.85	6.53E-03
23	rs17421	153225634	rs5987175	ADD	European	1.30	1.05E-06
23	rs13397	153248248	rs5987175	ADD	European	1.34	4.38E-07
23	rs1059702	153284192	rs5987175	ADD	European	1.32	2.20E-07
23	rs5987175	153153839	rs17421	ADD	European	1.02	6.90E-01
23	rs5987175	153153839	rs13397	ADD	European	1.02	7.25E-01
23	rs5987175	153153839	rs1059702	ADD	European	1.01	7.54E-01

Supplementary Table 6. The list of SNVs used in Supplementary Figure 3A, from left to right. The LD pattern (r^2) was calculated by the GWAS data used in the current study. Both r^2_Asian and r^2_EUR indicate the LD with rs13440883.

SNP	position	r^2_EUR	r^2_Asian
rs7877051	53071117	0.861689	0.498914
rs12399047	53071395	0.861566	0.497459
rs12400146	53071436	0.946979	0.692429
rs12399101	53071739	0.861689	0.498914
rs12399506	53072170	0.857985	0.482277
rs12400638	53072247	0.861689	0.500104
rs138701413	53072485	0.0604902	0.14709
rs73208441	53072602	0.861689	0.500244
rs73208442	53073113	0.881332	0.510207
rs13440965	53073712	0.0639324	0.156498
rs112815541	53074480	0.884532	0.511242
rs112413992	53074894	0.884532	0.511504
rs7886006	53076374	0.885273	0.510429
rs7880612	53076716	0.885273	0.508845
rs73634274	53077071	0.0630602	0.130132
rs113951189	53077747	0.894437	0.59347
rs73634275	53077998	0.0614544	0.147077
rs73634276	53079845	0.0623265	0.151298
rs55850908	53081414	0.987087	0.992071
rs59180338	53081558	0.987087	0.992072
rs67791620	53081814	0.976957	0.96523
rs67450314	53081970	0.98554	0.992044
rs12392162	53082363	0.986654	0.992059
rs12007081	53084492	0.987454	0.994511
rs12013318	53084568	0.991464	0.995301
rs7473078	53084799	0.987454	0.994514
rs67457527	53085169	0.987454	0.994521
rs73208449	53085633	0.892317	0.759438
rs145795871	53086455	0.0626712	0.157675
rs12389147	53086749	0.983831	0.971481
rs12390489	53087476	0.987081	0.996143
rs67171574	53087835	0.990739	0.996147
rs183981514	53089211	0.982055	0.994541
rs189101417	53089213	0.981784	0.994534
rs10855213	53090536	0.992973	1
rs73634281	53095639	0.993692	0.999239

rs67733752	53096176	0.993692	0.999239
rs28585589	53098061	0.904899	0.769299
rs111655908	53098327	0.904899	0.769213
rs112167847	53100196	0.906056	0.770618
rs12389413	53100590	1	0.999239
rs73634282	53101228	0.997386	1
rs138457679	53101263	0.0641387	0.168568
**rs13440883**	**53101477**	**1**	**1**
rs7881236	53101684	0.899629	0.757807
rs7883255	53101934	0.987427	0.996816
rs7473421	53102956	0.983918	0.995967
rs12388258	53105196	0.938446	0.990695
rs17850567	53105890	0.860489	0.741897
**rs11091720**	**53106865**	**0.938435**	**0.991546**
rs56157110	53107941	0.935763	0.990624
rs7060542	53109075	0.936104	0.991478
rs12399509	53110568	0.854483	0.638715
rs183116083	53110747	0.92113	0.988417
rs7885663	53114327	0.825134	0.810891
rs12400540	53115705	0.767558	0.790158
rs7891342	53115858	0.767735	0.78158
rs7891374	53115909	0.767804	0.779391
rs111433517	53116730	0.770995	0.480601
rs5933619	53120346	0.767804	0.777532
rs5933603	53124816	0.767804	0.759586
rs5978191	53126941	0.768242	0.74213
rs5978192	53126952	0.767559	0.74213

Supplementary Figures

Supplementary Figure 1. QQ plot for the cross-population X chromosome meta-analysis data. The X-axis is the expected association *P* value ($-\log_{10}P$), and the Y-axis is the observed association *P* value ($-\log_{10}P$). The left panel (black) includes all of the SNPs available on X chromosome, and right panel (red) excludes all of the known X-linked SLE susceptibility loci and the SNPs within the range of ±200 kb.

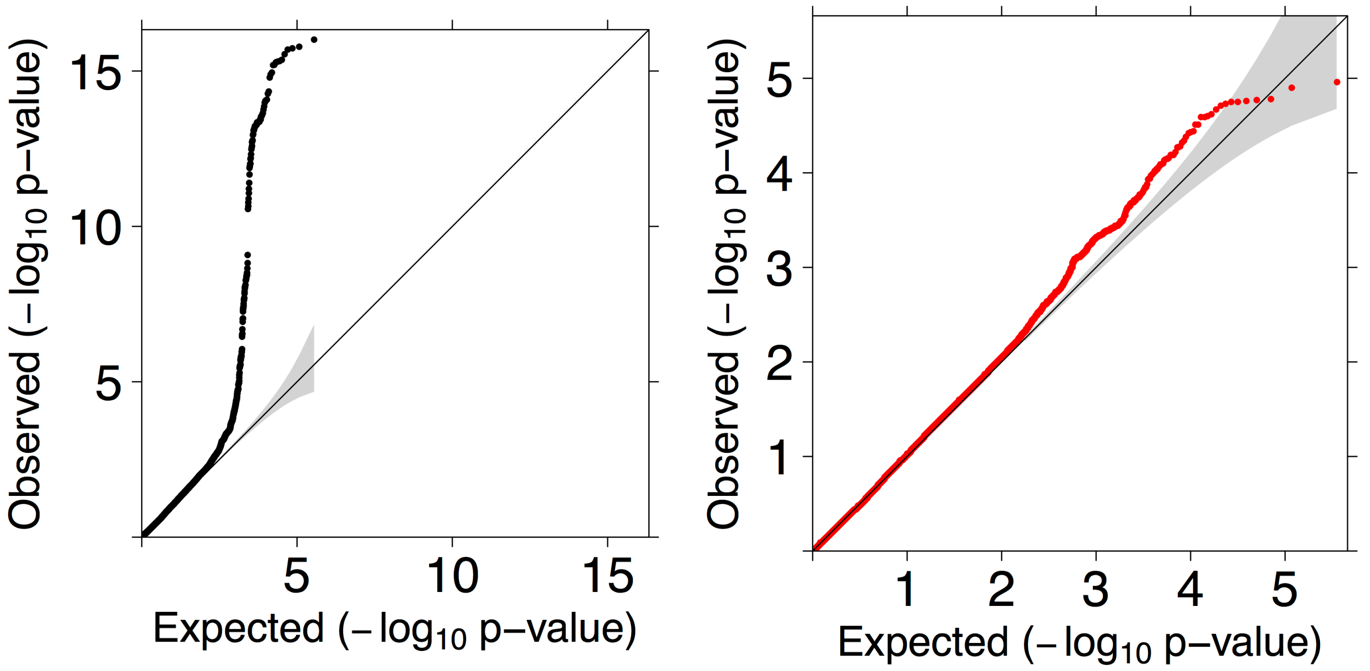
Observed (−log10 p−value)
15
10
5
5
10
15
Expected (−log10 p−value)
Observed (−log10 p−value)
5
4
3
2
1
1
2
3
4
5
Expected (−log10 p−value)

Supplementary Figure 2. The LocusZoom Plots showing association significance (-log$_{10}$*P*) and local LD (r^2) for the region around rs13440883 (±200kb). Validated SNP rs13440883 is labeled in purple.

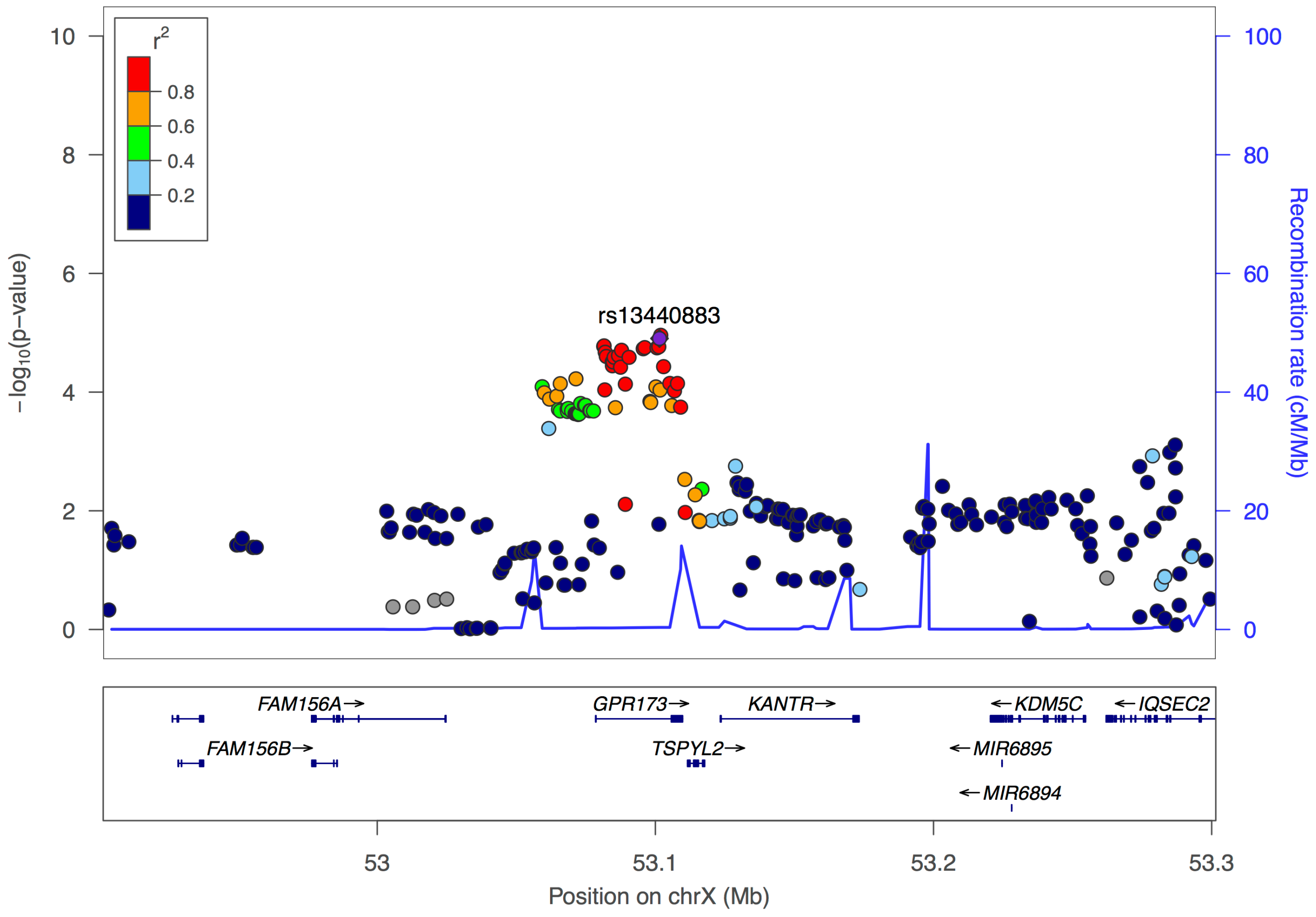

r2
0.8
0.6
0.4
0.2
rs13440883
−log10(p−value)
Recombination rate (cM/Mb)
FAM156A→
FAM156B→
GPR173→
KANTR→
TSPYL2→
←KDM5C
←IQSEC2
←MIR6895
←MIR6894
53
53.1
53.2
53.3
Position on chrX (Mb)

Supplementary Figure 3. Identification of functional risk-associated SNV shared between Europeans and Asians. (A) is the functional annotation of the SNPs around rs13440883 (± 30kb). "LD EUR" is the LD pattern of European and "LD Asian" is the LD pattern of Asian, while "LD merge" is the composite of "LD EUR" and "LD Asian". The green filled squares correspond to rs13440883, and green outlines indicate the SNVs in high LD ($r^2 > 0.8$) with it in both Asian and European population. The SNV list could be found in Supplementary Table 6. Functional annotations of K562 and GM12878 cell lines were extracted from ENCODE[23] project. (B) and (C) are the volcano plots for the IGR results of rs13440883 and rs11091720, plotted with the TF(s) overlapping the corresponding SNV, and the color schemes are the same as used in (A). The area of the circle is proportional to the maximum average signal intensity of the two alleles.

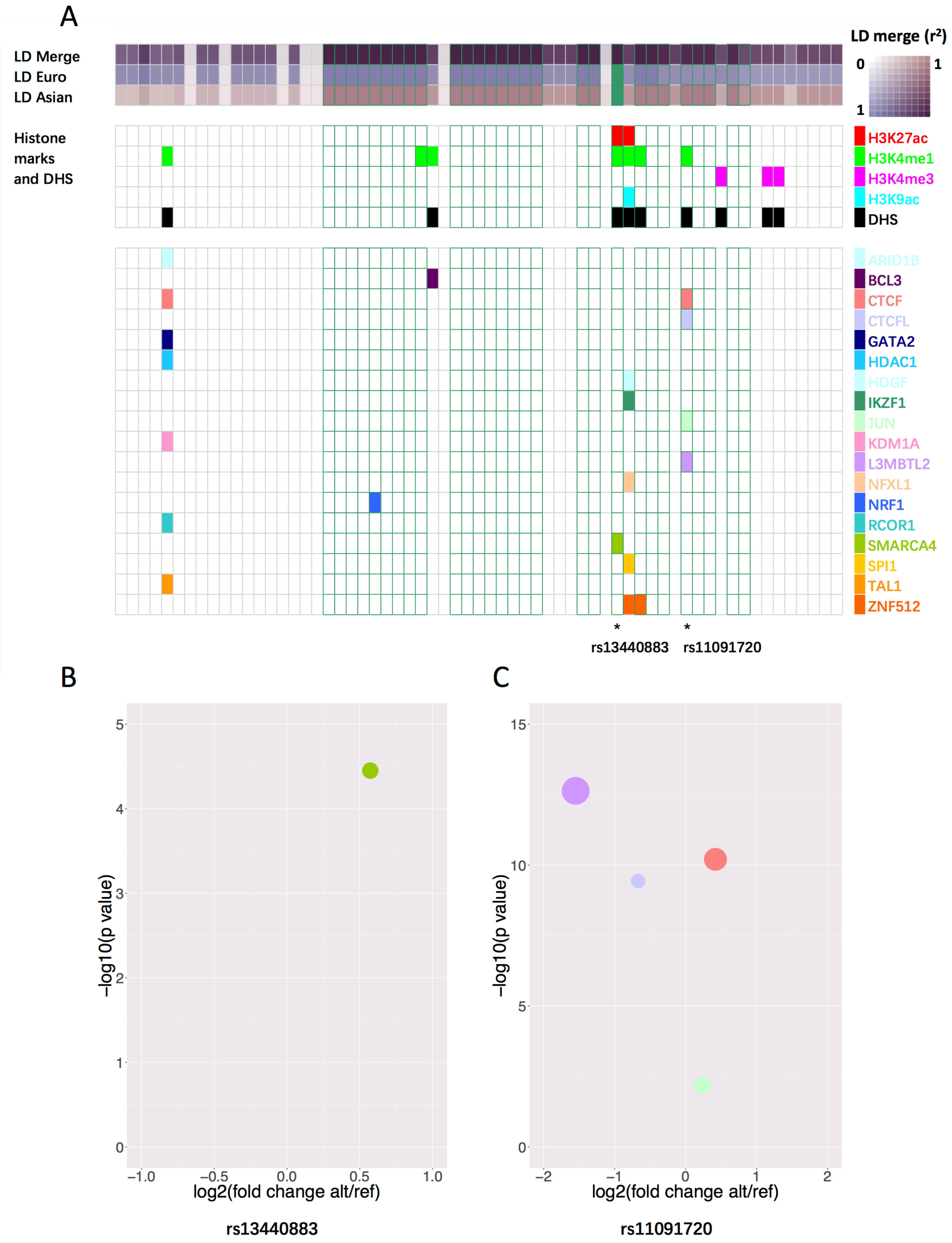
A
LD Merge
LD Euro
LD Asian
LD merge (r²)
0
1
1
Histone marks and DHS
H3K27ac
H3K4me1
H3K4me3
H3K9ac
DHS
ARID1B
BCL3
CTCF
CTCFL
GATA2
HDAC1
HDGF
IKZF1
JUN
KDM1A
L3MBTL2
NFXL1
NRF1
RCOR1
SMARCA4
SPI1
TAL1
ZNF512
*
*
rs13440883
rs11091720
B
−log10(p value)
log2(fold change alt/ref)
rs13440883
C
−log10(p value)
log2(fold change alt/ref)
rs11091720

Supplementary Figure 4. LD patterns (r^2) of the risk-associated SNPs ($P_{meta} < 1 \times 10^{-8}$) in *L1CAM-MECP2* region, plotted by the GWAS data used in the current study. From top to bottom are: the annotation of this region, the LD pattern of Anhui population, the LD pattern of Hong Kong population, and the LD pattern of European population. The suggested independent SNP rs5987175 is labeled in red and the top SNPs in other blocks are labeled with different color. LD blocks were indicated by blue outlines.

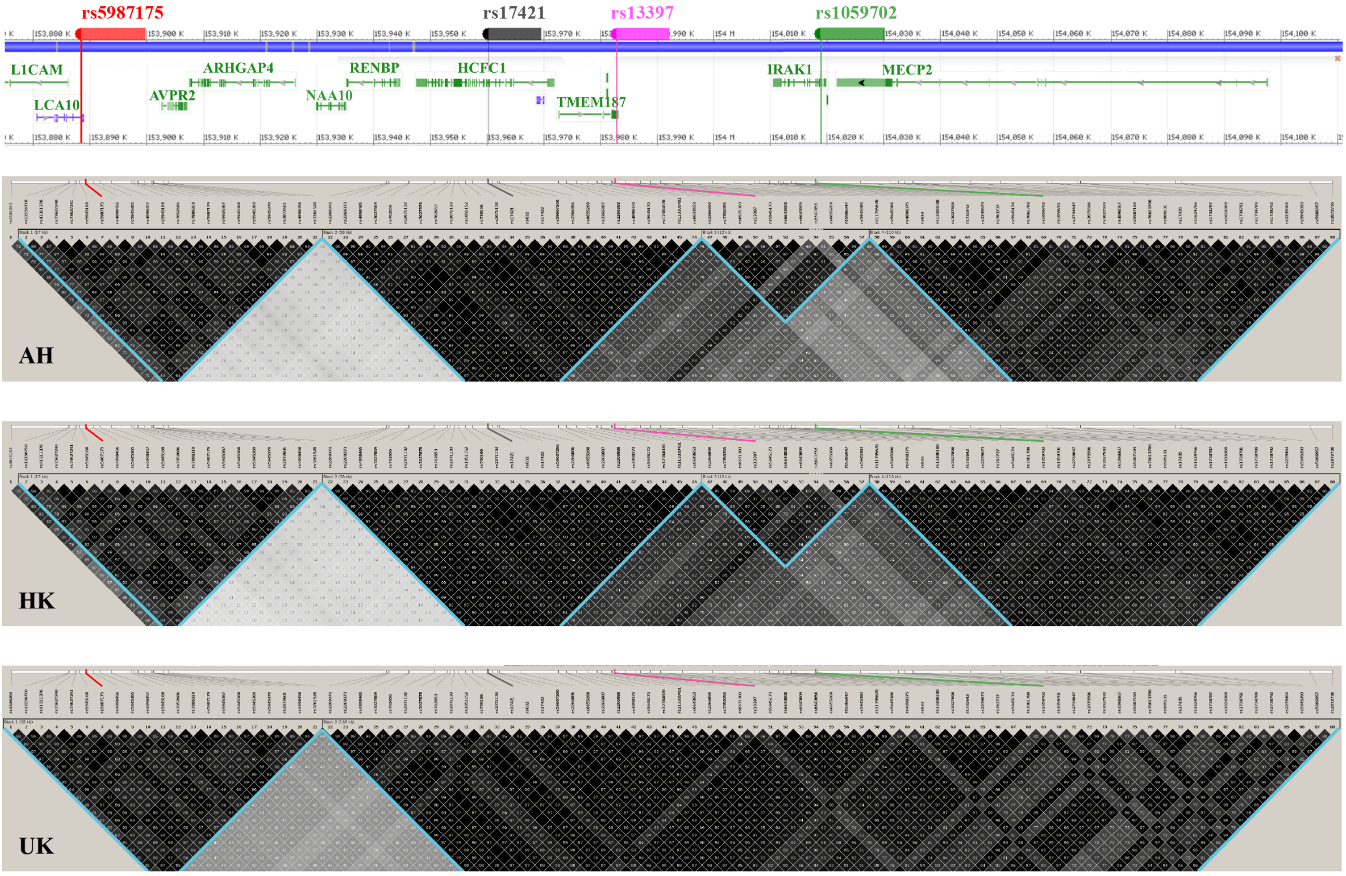
rs5987175
rs17421
rs13397
rs1059702
L1CAM
LCA10
AVPR2
ARHGAP4
NAA10
RENBP
HCFC1
TMEM187
IRAK1
MECP2
AH
HK
UK
